# Terahertz polarimetric imaging of biological tissues: Monte Carlo modeling of signal contrast mechanisms due to Mie scattering

**DOI:** 10.21203/rs.3.rs-3745690/v1

**Published:** 2023-12-14

**Authors:** Kuangyi Xu, M. Hassan Arbab

**Affiliations:** 1 Department of Biomedical Engineering, State University of New York at Stony Brook, Stony Brook, New York 11794, USA

## Abstract

Many promising biomedical applications have been proposed for terahertz (THz) spectroscopy and diagnostic imaging techniques. Polarimetric imaging systems are generally useful for enhancing imaging contrasts, yet the interplay between THz polarization changes and the random discrete structures in biological samples are not well understood. In this work, we performed Monte Carlo simulations of the propagation of polarized THz waves in skin and adipose tissues based on the Mie scattering from intrinsic structures, such as hair follicles or sweat glands. We show that the polarimetric contrasts are distinctly affected by concentration, size and dielectric properties of the scatterers, as well as the frequency and polarization of the incident THz waves. We describe the experimental requirements for observing and extracting these polarimetric signals due to the low energy and small angular spread of the back-scattered THz radiation. We analyzed the spatially integrated Mueller matrices of samples in the normal-incidence back-scattering geometry. We show that the frequency-dependent degree of polarization (DOP) can be used to infer the concentrations and dielectric contents of the scattering structures. Our modeling approach can be used to inform the design of the imaging modalities and the interpretation of the spectroscopic data in future terahertz biomedical imaging applications.

## 1. Introduction

The 30^*th*^ anniversary of open source Monte Carlo (MC) codes for modeling light propagation in tissue was recently celebrated in 2022 [1]. Over this period, MC models describing photon transport in biological tissues have been used to aid the design of diagnostic devices and to explain the observed phenomena. Examples of instrumental designs informed by MC modeling include the source-detector separations [2], angular dependence [3], polarization-sensitive methods [4,5] and coherence-domain techniques [6,7]. Similarly, MC codes are used to predict the response of samples from their bulk optical properties [8] or by use of the inversion techniques [9,10].

During the same period, terahertz (THz) spectroscopy methods have also matured rapidly, which has facilitated the investigation of various biological testing and several *in vivo* and clinical imaging applications [11–14]. A favored approach in THz biomedical sensing is to extract the dielectric permittivity of the sample from their reflectance or transmittance [15–17]. Further information on sample composition can be gained by applying appropriate effective media models, which relate the THz dielectric spectrum to the fractions and physical parameters of the presumed components. In particular, there has been significant success in the analysis of water content of the tissues [18–21], owing to the strong THz absorption by both bond and free water molecules. Still, this approach only provides a homogeneous solution, i.e., the loss of THz radiation in a spatial area of the sample is described using the Fresnel’s and Beer’s laws [22,23].

The presence of nonuniform dielectric structures can generally produce disturbance of light propagation in an otherwise homogeneous medium [24,25]. However, whether such a disturbance can measurably be confirmed in THz biomedical applications has remained as a point of concern in the scientific community, considering the strong dielectric absorption of the homogeneous medium and reduced scattering at longer wavelengths. Previously, such deviations from bulk optical response of a sample has been experimentally reported in biological tissue with existing THz systems. Feldman et al. have reported that the THz spectra of human skin can be notably affected by the eccrine sweat glands [26], given that active glands have high proton conductivity and are thus analogous to helical antennas [27]. Separately, we have found that the THz response of rat skin with burn injuries is not only accounted by the level of water contents, but also correlates with the density of discrete structures recognized in the sample histology [28–30]. In addition to skin tissue, the idea of combining hydration analysis and correlation to sample non-uniformity have also been proposed for other types of tissue [31,32]. The potential of these investigation comes from two aspects: (1) The presence of nonuniform structures (either intrinsic or extrinsic) opens up new modalities for THz sensing; (2) These nonuniform structures often play an important role in biological processes such as wound healing, diagnostics and therapy [33].

With the enhancement in SNR and expansion of measurement capabilities, new paradigms are emerging in THz biomedical sensing based on alternative contrast mechanisms due to complex heterogeneous, birefringent and stratified sample profiles. For instance, recent THz ellipsometric measurements of *in vivo* human skin has revealed anisotropic cellular structure of the stratum corneum, birefringence and occlusion effects as well as sensitivity of hydration measurements to surface scattering [34–36]. Although such scattering effects can be mitigated using signal processing methods [37,38], they can also be diagnostically useful and can be measured using highly-accurate and calibrated broadband polarimetry techniques [39,40].

Our objective in this work is to develop predictive models to relate the nonuniform structures in biological samples to THz scattering measurements, and to explain such observations. Similar to the optical regime [41], several approaches can be implemented for THz scattering measurements: (1) angular-resolved magnitude measurements, (2) broadband spectroscopic measurements at a fixed angle, and (3) measurements of coherence or polarization state of the light beam [42]. Angular or frequency dependent THz systems can readily characterize the scattering in granular materials [43–46]. Given the abundance of overlapping absorption modes in biological samples [47], however, it becomes challenging to separate the contribution of scattering from featureless absorption or measurement noise. Meanwhile, THz polarization imaging can potentially provide better contrasts in tissue delineation [48, 49]. Still, unlike examples in the optical regime demonstrated by polarization gating [50], selective probing of superficial tissue structures has not been reported in THz frequencies.

In this paper, we preformed MC simulations of the THz radiative transfer in biological tissues as the function of detecting position, frequency and polarization of THz sources. Previously, Ney et al. used similar MC codes to investigate the THz Mie scattering in skin induced by extrinsic nanoparticles [51], whereas here we considered the intrinsic structures that have comparable dimensions to the THz wavelengths. Upon the introduction of scattering particles, we found a diffuse component in the THz reflection from skin, which exhibit different degree of polarization (DOP) as the functions of source frequencies, or the concentration and inside contents of particles. Our approach is motivated by future application of our polarization-sensitive handheld scanner [52,53] for *in vivo* and longitudinal diagnosis of skin burns [54–56]. Therefore, this paper is focused on similar experimental conditions of instrument and sample type and scattering particles. We hope that the widespread adoption of the simulation algorithms introduced in this work will also be useful for other applications of THz biophotonics [57].

## 2. Methods

In this section, we provide a detailed description of the numerical models needed for simulating the THz beam propagation in tissue, including the tissue models, the MC algorithms, and data analysis approaches to extract the polarimetric signal contrasts. We have adopted two separate algorithms to model the propagation of THz photons in stratified layers and to analyze the polarization of THz waves after scattering by discrete skin structures. The former issue is addressed by the Monte Carlo Multi-Layered (MCML) codes provided by Wang et al. [58,59], which has gained great success in the optical regime for modeling tissue structures, e.g., in skin and eyeball [60]. The latter is solved by the Meridian Plane method [5,59], which we will refer to as the Polarized-Light MC codes. Further, we made modifications to the original MC codes based on the theory of Mie scattering in absorbing media as described below.

### 2.1. Modeling of the skin structures

**Fig. 1.**
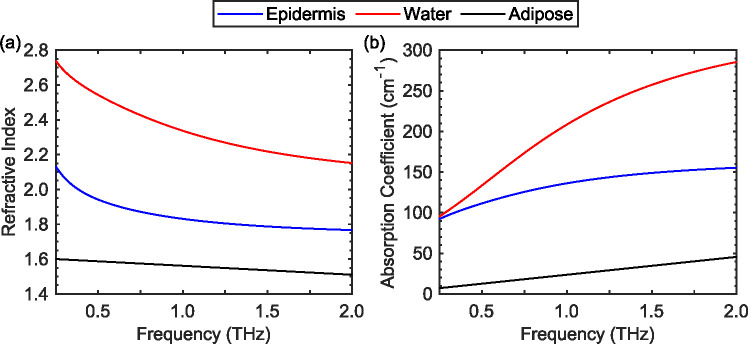
The refractive index (a) and absorption coefficient (b) of epidermis, water and adipose tissue in the frequency range between 0.1 and 2 THz.

Due to the strong absorption by water, THz radiation has a penetration depth around 0.1–0.5 mm into the skin [12,61], which is less than the typical thickness of the dermis. Therefore, a three-layer [26,51] or two-layer [62,63] approximation is often adopted for simulating the THz spectra of skin. Figure 1 shows the dielectric functions of the epidermis and bulk water calculated using the Double-Debye dispersion model [15]. The THz properties of the dermis tissue are generated by assuming the Maxwell-Wagner mixing law of water and the dry skin components, following the approach described in Ref. [51,64]. For a comparison study, we also considered the adipose tissue as a representative case for less lossy media, since the lipids attenuate THz radiation less strongly than skin. Yanina et al. have reviewed THz measurements of adipose tissue [65], and we have adopted the one reported in Ref. [66], as plotted in Fig. 1.

Another aspect of our skin model is to incorporate discrete particles with size and density resembling observed conditions. If the discrete particle with a radius, r, is much smaller than the wavelength (λ), i.e., the size parameter x=2πr/λ≪1, the resultant scattering will be in the Rayleigh regime. For example, between 0.1 and 2 THz, the size parameter x for a 10 *μ*m diameter cell varies from 0.01 to 0.21. Although large cells may still exhibit Mie scattering at higher THz frequencies, we have focused on skin appendages, which are more likely to induce measurable signal contrasts. The scattering structures proposed by previous THz studies, including sweat glands [62], and hair follicles [67] having an average diameter and length between 100 and 500 *μ*m [30], are represented by “equivalent spheres” for simplicity [68], which give rise to the size parameter larger than 0.2, in the frequency range between 0.1 and 1 THz [45]. The concentration of particles were calculated from histological sections, and two dielectric constant values were simulated to resemble the skin with both empty (air) and filled (water) sweat glands.

### 2.2. Monte Carlo simulation algorithms

There are many open-source resources and tutorials available for MC simulation of radiative transport [60, 69], including codes and manuals that are readily accessible to the scientific community [59]. Here we focus on providing a brief background necessary for accurate modeling of the skin appendages in the THz frequency range. When a new photon packet is launched in a MC simulation, it travels for a distance of s before experiencing an interaction, such as absorption or scattering. The sampled value of s is related to the optical properties of a turbid medium by,

(1) $$s=-ln\;(\xi )/\mu_{e},$$

where ξ is a random number between 0 and 1, and $\mu_{e}$ is the extinction coefficient of the medium. In general, these interactions involve either scattering or absorption. The impact of absorption is described by the albedo, α, of the medium:

(2) $$\alpha =\mu_{s}/\mu_{e},$$

where,

(3) $$\mu_{e}=\mu_{s}+\mu_{a},$$

and $\mu_{s}\left(\mu_{a}\right)$ is the scattering (absorption) coefficient of the medium. In each scattering event, the weight of photon (denoting its energy) drops from W to W×α.

Subsequently, it is necessary to define the trajectory of the photon after each scattering event. In the MCML code, the photon trajectory is determined based on the Henyey–Greenstein (HG) phase function [58],

(4) $$P_{HG}\left(\cos\theta \right)=\frac{1}{2}\frac{1\;-\;g^{2}}{{\left(1\;+\;g^{2}\;-\;2g\cos\theta \right)}^{\frac{3}{2}}},$$

which empirically describes the angular pattern of scattering. $P_{HG}$ depends on only the asymetric factor g, which is the average cosine of the scattering angle θ.

In the Polarized-Light MC codes, this step is instead based on the Mie theory, which derives the relation between incident ($E_{i}$) and scattered ($E_{s}$) field amplitudes as,

(5) $$\left[\begin{array}{c} E_{s}^{⊥}\\ E_{s}^{∥} \end{array}\right]\propto \left[\begin{array}{cc} S_{1} & 0\\ 0 & S_{2} \end{array}\right]\left[\begin{array}{c} E_{i}^{⊥}\\ E_{i}^{∥} \end{array}\right].$$

Eq. 5 uses the Jones calculus based on the perpendicular and parallel components to the scattering plane. The scattering matrix only consists of two elements, $S_{1}(\theta )$ and $S_{2}(\theta )$, due to the spherical symmetry of particle [70]. The Mueller calculus transform of Eq. 5 is usually adopted so that the operation only involves real values, which is given by,

(6) $$\left[\begin{array}{c} I_{s}\\ Q_{s}\\ U_{S}\\ V_{s} \end{array}\right]\propto \left[\begin{array}{cccc} S_{11} & S_{12} & 0 & 0\\ S_{12} & S_{11} & 0 & 0\\ 0 & 0 & S_{33} & S_{34}\\ 0 & 0 & -S_{34} & S_{33} \end{array}\right]\left[\begin{array}{c} I_{i}\\ Q_{i}\\ U_{i}\\ V_{i} \end{array}\right].$$

**Fig. 2.**
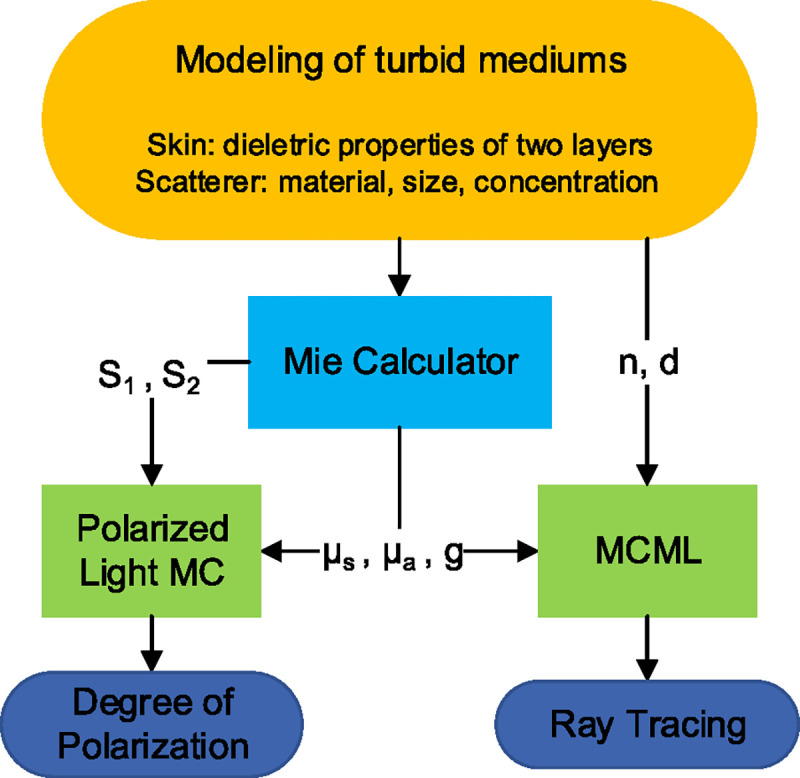
Flowchart of the input and output data in the steps of numerical simulation for THz radiation transport in skin model.

In addition to the new trajectory, Eq. 6 also determines the polarization change of the photon after each scattering event, which forms the foundation of the Polarized-Light MC codes. On the other hand, the MCML codes make fewer assumptions on the nature of turbid media, and also account for the Fresnel reflections which occur in the stratified structures. We have applied the two methods for different purposes, as described in the logical flowchart of our simulation presented in Fig. 2. While some authors have combined the merits of both methods to develop their own codes [51,71], alternatively we have used the two open-source codes [59] without large modification. Meanwhile, we have incorporated a Mie calculator to generate the required input parameters based on media models, which will be introduced in the following section.

### 2.3. Mie scattering in absorbing host media

As introduced earlier, the polarized light MC codes rely on the solutions to Mie scattering as input parameters, in particular the complex functions $S_{1}$ and $S_{2}$. Meanwhile, the MCML codes only depend on $\mu_{s}\left(\mu_{a}\right)$ and g, which are often obtained from direct measurements in optical systems. In the THz regime, however, such measurements on the scattering effects in biological tissues are still lacking or inconclusive. Therefore, we need to simulate these properties under Mie scattering assumptions as well.

Conventionally, the solutions to Mie scattering have assumed the host medium to be non-absorbing, i.e., its refractive index $n_{h}$ being a real value. This has been a reasonable approximation in optical regime, where the scattering effects of tissues are usually much stronger than absorption effects. On the contrary, THz waves experience strong absorption due to the water content, and less scattering as the wavelengths become much larger than that of visible light. Also, neglecting the imaginary part of $n_{h}$ would introduce significant error to the boundary conditions at the particle-medium interfaces. Therefore, it is necessary to modify the solutions to Mie scattering to account for the absorbing host media. The derivation of such modifications have been presented in the literature [72–74]. Here, we will briefly introduce key relationships for completeness and to avoid ambiguity in defenition of relevant parameters.

In absorbing host media, absorption occurs in both the host medium and the particle-medium boundary. We have adopted the formulas proposed by Yang et al. [73], where the label p denotes the properties of particle, h denoted the properties of host medium. The extinction of a photon packet before a scattering event occurs is expressed by,

(7) $$W=W\alpha \exp\left(-s\mu_{a,h}\right).$$

The absorption by host medium is a continuous reduction of W along the optical path, whereas the absorption of particle is still given by α, which is

(8) $$\alpha =\mu_{s,p}/\mu_{e,p},\;\mu_{e,p}=\mu_{s,p}+\mu_{a,p}.$$

In the modified Mie theory, the scattering (absorption) coefficients of the particle are derived as,

(9) $$\mu_{\text{s},\text{p}}=\frac{2\pi \rho }{{\left|n_{\text{h}}\right|}^{2}k_{0}^{2}}\sum_{j=1}^{\infty }\left(2j+1\right)\left({\left|a_{j}\right|}^{2}+{\left|b_{j}\right|}^{2}\right),$$

**Fig. 3.**
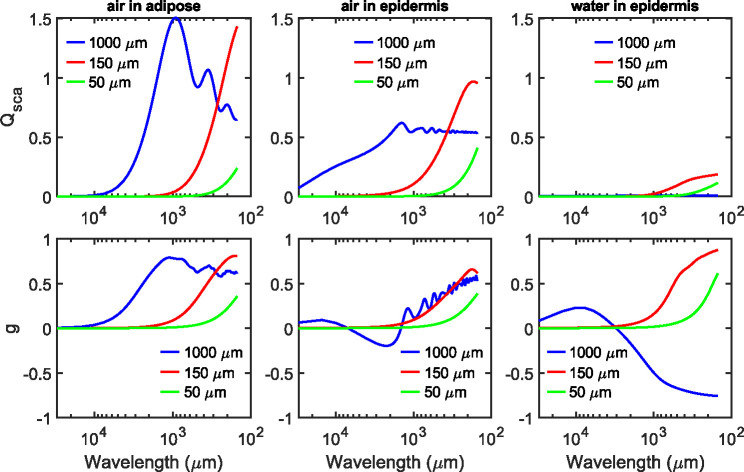
The scattering efficiency $Q_{\text{sca}}$ and asymmetric factor g for a spherical particle of 50, 150 or 1000 *μ*m diameter, with different particle contents and host media.

(10) μa,p=2πρRe(nh)k02Im[(∑j=1∞Aj)/np],

where $n_{h}\left(n_{p}\right)$ is the complex refractive index of the medium (particle), $k_{0}=2\pi /\lambda_{0}$ is the vacuum wave vector, ρ is the number density of particles. The complex polynomial $A_{j}$ are given by,

(11) $$A_{j}=\left(2j+1\right)[{\left|c_{j}\right|}^{2}\psi_{j}\left(z\right)\psi_{j}^{\prime *}\left(z\right)-{\left|d_{j}\right|}^{2}\psi_{j}^{\prime }\left(z\right)\psi_{j}^{*}\left(z\right)],$$

(12) $$z=n_{p}k_{0}r,$$

where $a_{j},$ $b_{j},$ $c_{j}$, and $d_{j}$ are the Mie coefficients, and $\psi_{j}(z)$ is a Riccati–Bessel function. The definitions of these parameters remain the same as those defined for non-absorbing media [70], except that $n_{h}$ is replaced with a complex value. Hence, we modified the Mie calculator for absorbing media by adapting the Matlab codes provided by Mätzler [75]. We have applied the calculator for physical conditions that tend to occur in skin samples and compared the results with theoretical expectations as validation of these calculations. Figure 3 presents the calculation results for three sizes of water-filled or air voids structures inside the epidermis or adipose tissue in the wavelength range from 150 to 30000 *μ*m (0.01–2 THz).

The scattering efficiency $Q_{\text{sca}}$, as shown in the first row, is the ratio of scattering cross-section of single particle to its geometrical area. We see that $Q_{\text{sca}}$ in all cases is significant when the particle diameter is comparable to or greater than the wavelength, which is the feature of Mie scattering. As the size parameter tends to infinity, $Q_{\text{sca}}$ converges to 2 for air voids in an non-absorbing medium, while it converges to 0.5 for absorbing medium [74]. Such a difference can be seen in Fig. 3 between the adipose and epidermis tissue. Note here that adipose has a small but non-negligible absorption in the host medium, which results in the deviation from this theoretical limit of 2 for non-absorbing scattering efficiency $Q_{\text{sca}}$. If both particles and host media are absorbing, $Q_{\text{sca}}$ would converge to zero instead, which can be seen in the case of water in epidermis. Asymmetric factorg, introduced in Eq. 4, usually rises toward 1 as the size parameter increases. However, gconverges to −1 when simulating extremely large absorbing particles, as seen by the blue curve in the case of water in epidermis. The physical interpretation of this trend is that the forward scattering is strongly attenuated by the absorption in particles.

## 3. Results

### 3.1. Distribution of the THz radiation energy

**Fig. 4.**
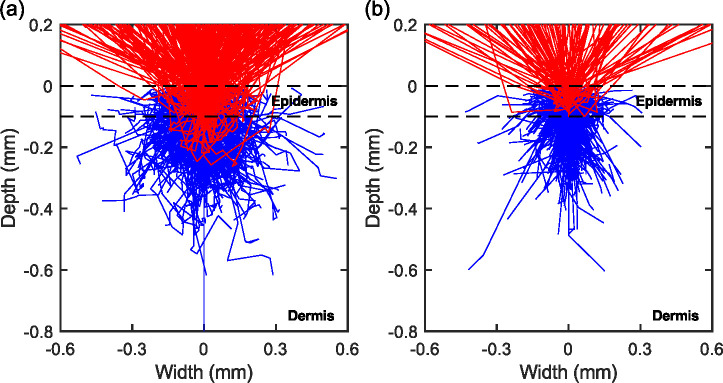
Simulation of the propagation of photons when spherical scatterers consist of (a) air or (b) water are embedded inside skin. (a) and (b) both assume radiation frequency of 0.6 THz, particle diameter of 150 *μ*m and mean inter-particle distance of 200 *μ*m.

To study the propagation of THz radiation inside skin, we start with the MCML code, as outlined in Fig. 2, which generates the traces of photon packets based on the bulk optical properties of different tissue layers. Fig. 4 shows a point spread function simulation of photon trajectory at 0.6 THz, when the dielectric function of the skin appendages are varied in parts (a) and (b), but under the same assumption of host media, i.e., the thickness (d), refractive index (n) and absorption coefficient $\left(\mu_{a,h}\right)$ of the epidermis and dermis layers are the same. We assumed scattering particles of 150 *μ*m diameter with the concentration of 125 mm^−3^, based on the approach mentioned in section 2.1. using representative skin histology. The dielectric properties of the particles are chosen to represent the two extreme cases of scattering efficiency, i.e., air and water. The former case represents empty sweat glands or hair follicles, while the latter corresponds to structures like filled sweat glands. In both scenarios simulated in Fig. 4, the likelihood of photon absorption (blue lines) are higher than escaped photons upon scattering (red lines). Also, it is evident that the majority of reflected photon packets have only travelled inside the epidermis. Here a thickness of 100 *μ*m was assumed for epidermis. Since the epidermal thickness varies at different body sites and between individuals [76], there will be more photon packets reaching the dermis if a thinner thickness value is used. Comparing the scattering from air and water particles, the penetration depths appear to be similar, since the attenuation are largely contributed by the absorption in the host media $\left(\mu_{a,h}\right)$. Still, there are considerable differences in the spatial probability distributions of photons (i.e., the shape of the interaction volume). This is essentially a visual interpretation of the asymmetric factor g, whose values are previously calculated in Fig. 3. In other words, when g approaches to 1 a larger degree of scattering in the forward direction is specified. Another noticeable change in Fig.4(b) is that less photon packets have escaped from skin when the scattering particles are filled with water, which can be attributed to (1) the absorption due to these particles $\left(\mu_{a,p}\right)$ and (2) smaller mismatch between the refractive index of particles and host media (see Fig. 1). These results suggests that the complex refractive indices have been reasonably incorporated into our MC simulation.

**Fig. 5.**
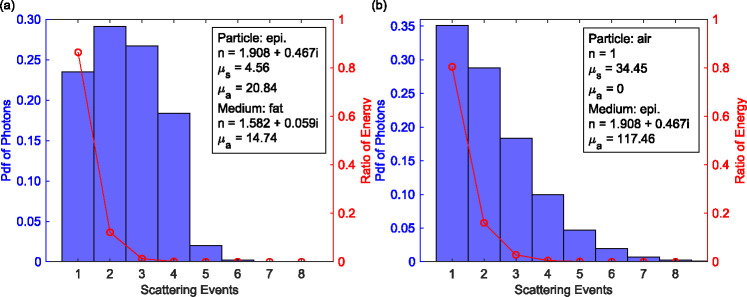
The distribution of scattered photons as the function of scattering events in the case of (a) epithelial spheres embedded in adipose, (b) air spheres embedded in epidermis. Inset: assumed optical properties of tissues at 0.6 THz.

Before continuing with the discussion of polarimetric signal contrasts, we need to make some clarifying comments regarding results shown in Fig. 4. First, we have considered a quartz slab above the skin in these simulations, which corresponds to a microscope slide for *ex vivo* samples or an optical window for *in vivo* imaging. In the THz regime, the refractive indices of glassy materials (e.g., quartz) are often comparable to or higher than that of skin. If we choose not to apply the quartz layer, the traces of photons change consequently due to total internal reflections of THz waves at the epidermis-air interface. Second, the ratio and spatial distribution of scattered intensities are different from what can be visually estimated from Fig. 4, since the weight information, W, is not carried by the ray traces. This information can be discerned, however, from Fig. 5, which shows the probability distributions of the exiting photons as the function of the number of scattering events. The probability distribution functions shown by the histogram (blue bars) suggest that the radiation at 0.6 THz experience different orders of scattering depending on the absorption of the host media. However, the weighted distributions of exiting photons (the energy ratio) reveal that the scattered intensity is mostly due to the single-scattering photons. The red curves show that there is also a minor contribution by the double-scattering photons, whereas the impact of higher-order scattering processes are generally negligible. Note that this conclusion is valid for both weak and high absorption biological tissues, as represented by adipose in Fig. 5(a) and epidermis in Fig. 5(b), respectively. Therefore, the predictions of a single-scattering model will be used to explain other characteristics of the simulation results, namely the polarimetric signatures of the tissue, which will be shown in the following sections.

### 3.2. Distribution of the THz polarization states

**Fig. 6.**
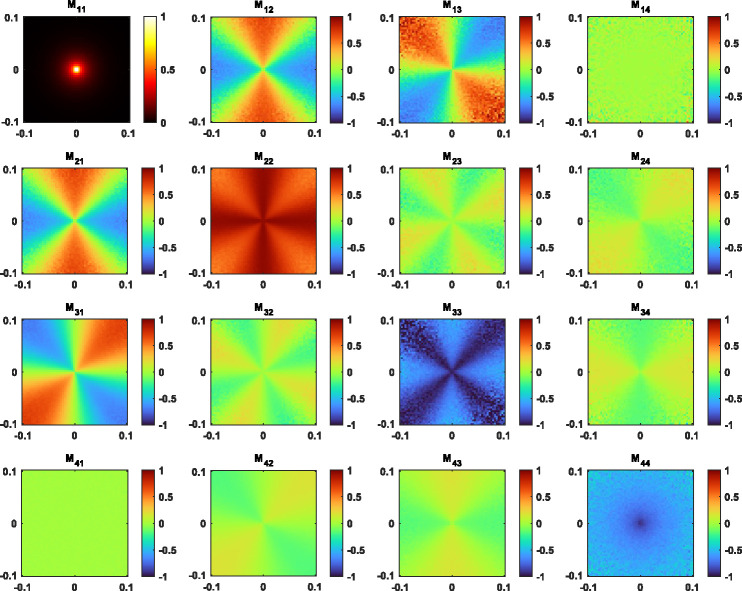
Maps of the normalized Mueller matrix in a 0.2 × 0.2 cm^2^ imaging area for 150 *μ*m epithelial spheres in adipose tissue, illuminated with a pencil beam at 0.6 THz.

In addition to the distribution of power, the polarization changes of THz waves occurred during propagation are investigated using the Polarized-Light MC algorithm, as described in Fig. 2 flowchart. This algorithm can generate the spatial maps of the four Stokes parameters (I,Q,U,V), while four independent polarization states of the incident light can be selected as the input values. Subsequently, the 4 × 4 combination of maps can be transformed into the Mueller matrix of the tissue, as shown in Fig. 6 with the normalized values. Our results that $m_{14}\;$and $m_{41}$ are close to zero regardless of the biological tissues, and that certain elements (e.g., $m_{22}$ and $m_{33}$) have symmetric patterns, similar to the results obtained in the optical regime [77,78]. However, it will be difficult experimentally to observe these spatial (angular) patterns with current THz systems, since most of the scattered radiation energy is distributed in a narrow imaging area (angular spread), as shown by the map of $m_{11}$. Instead, we use the pixel sums of these maps to simulate the Mueller matrix captured by an on-axis single-element detector. This matrix M for Fig. 6 is given by,

(13) $$M=6.7\times 10^{-3}\left[\begin{array}{cccc} 1.000 & 0.000 & 0.000 & 0.001\\ 0.000 & 0.837 & 0.000 & 0.000\\ 0.000 & 0.000 & -0.837 & 0.000\\ 0.000 & 0.000 & 0.000 & -0.689 \end{array}\right].$$

Here, we note that M is a diagonal matrix and it complies with the additional condition that

(14) $$m_{11}-m_{22}+m_{33}-m_{44}\approx 0,$$

which is in agreement with theoretical predictions resulted from assuming an ensemble of single scatterers each of them having a reciprocal Mueller-Jones matrix [79,80]. Therefore, this result is consistent with the PDFs shown in Fig. 5. When $m_{22}=-m_{33}$ is also satisfied, the Mueller matrix can be rewritten into a simple form, by substituting $m_{11}$ and $m_{22}$ with symbol $a_{1}$ and $a_{2}$, respectively, given by,

(15) $$M=\mathrm{diag}\left[a_{1},a_{2},-a_{2},a_{1}-2a_{2}\right]=a_{1}\mathrm{diag}\left[1,p,-p,1-2p\right],$$

where $p=a_{2}/a_{1}$ This legacy form of the Mueller matrix has been substantiated for the backscattering from non-chiral particles with a rotational symmetry in a cloud of particles [80,81]. This matrix can be decomposed as a uniform attenuator, i.e., parameter $a_{1}$, and a nonuniform partial depolarizer, i.e., diag[1, p, -p, 1–2p] [82]. Therefore, the polarization changes of the scattered fields $\left(E_{s}\right)$ are sufficiently described by the Degree of Polarization (DOP), defined as,

(16) $$DOP=\sqrt{Q^{2}+U^{2}+V^{2}}/I.$$

According to Eq. 15, the DOP of $E_{s}$ equals |p| when incident light $E_{i}$ is linearly polarized (LP), and equals |1–2p| when $E_{i}$ is circularly polarized (CP). The linear and circular DOP are hence correlated and their order depends on the range of p.

**Fig. 7.**
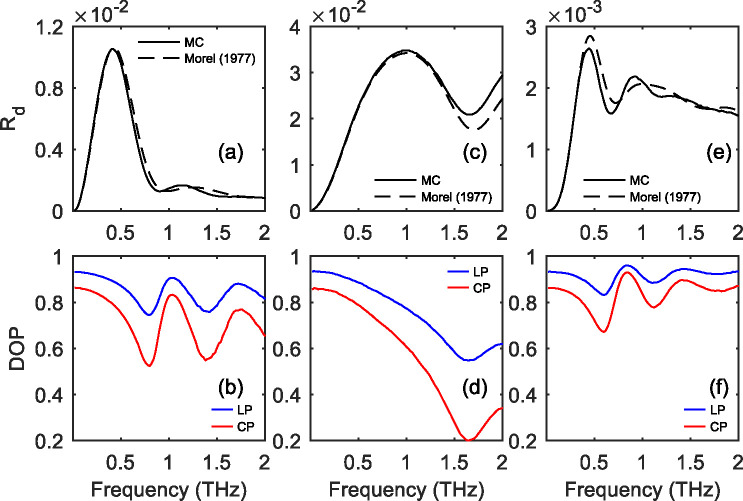
(a) Diffuse refletance $R_{d}$ and (b) degree of polarization (DOP) for the total THz radiation scattered by epithelial particles in adipose tissue. (c) and (d) are obtained for air voids in skin. (e) and (f) are obtained for water particles in skin. All simulations use the same particle diameter of 150 *μ*m and mean inter-particle distance of 200 *μ*m.

Figure 7 presents the DOP of THz waves obtained with the same particle size and density values as previously set in Fig. 4. In general, the linear and circular DOP show similar dependence on the THz frequency, because they are both determined by the the frequency dependence of p. In addition, our results show that pϵ(0.5,1)is valid under the three simulation conditions, leading to a higher DOP of LP than that of CP (p>2p-1>0). This is opposite to the usual outcome for the Mie scattering in tissues in the optical regime [83], where $\left|m_{33}\right|<\left|m_{44}\right|$. We attribute this obervation in the THz frequencies to the prevalence of single-scattering and the small spread of scattering angle θ, considering that singly scattered radiation stays fully polarized at θ=180°.

### 3.3. Concentration dependence of the scattering signal contrasts

In order to measure the signal contrast in DOP, we also expect the diffuse reflectance $R_{d}$ to be above the SNR threshold of the current THz polarimetric systems. As shown in Fig. 7, the diffused reflectance, $R_{d}$, given by the MC simulations is in good agreement with the following relation derived by Morel & Prieur [84],

(17) $$R_{d}=0.32\frac{b_{b}}{a\;+\;b_{b}},$$

where absorption coefficient a was approximated by $\mu_{a,h}+\mu_{a,p}$, and the backscattering coefficient $b_{b}$ was calculated from the integral of $S_{11}(\theta )$ (Eq. 6) in the backward hemisphere. As shown in Fig 3, the asymmetric factor g approaches to 1 as the frequency reaches 2 THz, indicating less scattering in the backward direction. The scattering anisotropy, together with the increasing absorption (a), explain why $R_{d}$ is diminished in the higher frequency region.

**Fig. 8.**
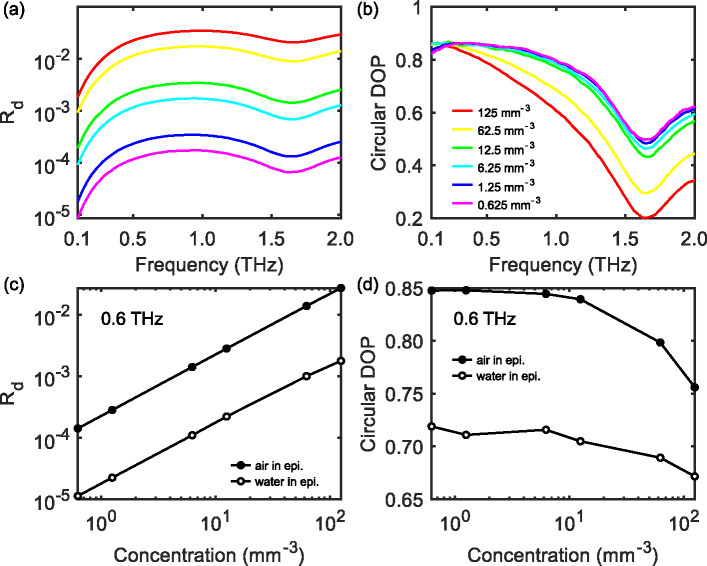
Variations of (a) the diffuse refletance $R_{d}$ and (b) the circular DOP in 0.1–2 THz as changing the concentration of air voids in the epidermis. (c) and (d) compare the concentration dependence of $R_{d}$ and circular DOP at a single frequency (0.6 THz) between the scatters made by air and water.

It can be inferred from Eq. 17 that $R_{d}$ is proportional to $b_{b}$ (when $b_{b}\ll a$) and thus to the particle concentration ρ. Figure 8(a) and (b) shows the variation of $R_{d}$ and circular DOP when diluting the concentration of air voids in the epidermis, with the red curves $\left(\rho =125{\;mm}^{-3}\right)$ representing the same data as those in Fig. 7(c) and (d). The decrease in $R_{d}$ are similar at different frequencies, and a linear relation between $R_{d}$ and concentration can be confirmed at 0.6 THz (in Fig. 8(c)). Meantime, the circular DOP approaches to unity as the concentration decreases (in Fig. 8(d)). This figure provides a reference for estimating the SNR requirement of THz polarimetric systems in order to characterize the scattering structures in skin.

## 4. Discussion

Comparing the results of the THz radiation transfer model in skin with adipose tissue, the absorption in our models rapidly increases in the presence of water content in either the scattering particles or host media, leading to low values of the single-scattering albedo α. This broadband behavior can be observed over the range of 0.1–2 THz, over which both the scattering and absorption coefficients rise with higher frequencies. Consequently, the low albedo increases the amount of single scattering relative to multiple scattering. This relation is rather straightforward in the MC simulation. The weight of photons drop by a fixed ratio of α during each interaction, therefore the energy ratio of exiting photons having undergone 1, 2, . . . , n scattering events decay geometrically with the orders [85,86]. Such geometric series can be seen in Fig. 5, with α=0.11 in (a) and α=0.23 in (b). The discrete structures of interests can be represented with different particle shape, size or concentration, however, it is unlikely to cause dramatic increase in α and hence the simulation results would still favor single scattering.

At θ=180°, the single-scattering by spherical particles stay as fully polarized [70]. With normal incidence, the specular reflections by the layered interfaces also propagate in this direction. Therefore, the THz waves reflected by skin will be measurably depolarized only if sufficient off-axis scattered rays are collected by the detector. In section 3.2 we investigated the pixel sums in an area wider than the point spread function, simulating a 2*π* collection solid angle. The contrast in DOP will be more significant if the off-axis components can be selectively resolved by the detector, which is yet a challenging task since the angular spreads of the THz reflection are rather narrow. Lastly, the angular patterns of scattering are also affected by assuming oblique incidence or non-spherical particles, which is worth further investigation.

## 5. Conclusion

We have preformed MC simulations for polarized THz radiation transfer in the absorbing host media doped with spherical particles, representing the potential Mie scattering by the accessory structures in biological tissues. The tissue models exhibit low single-scattering albedo when water contents are introduced in either the particles or host media, leading to the prevalence of single scattering in the scatterd THz radiation energy. In the geometry of normal incidence and total backscatter, the skin models behave as a uniform attenuator with a nonuniform partial depolarizer. We have investigated the diffuse reflectance and DOP of scattered THz waves as a function of frequency with different source polarization states, and in different conditions of the concentration and dielectric constant of scattering particles. These results suggest alternative signal contrasts that are distinctly associated with the skin appendages, and also provide estimation of the SNR requirement for observation of these effects using THz polarimetric instruments. Future work may include validation of the simulation results with controlled measurements using phantoms or *ex vivo* tissue samples. The simulation models and algorithms developed in this paper will enabale and expedite the discovery of future applications of THz biophotonics.

## Data Availability

Data underlying the results presented in this paper are not publicly available at this time but may be obtained from the authors upon reasonable request.
